# Functional Heterogeneity within the Default Network during Semantic Processing and Speech Production

**DOI:** 10.3389/fpsyg.2012.00281

**Published:** 2012-08-13

**Authors:** Mohamed L. Seghier, Cathy J. Price

**Affiliations:** ^1^Wellcome Trust Centre for Neuroimaging, Institute of Neurology, University College LondonLondon, UK

**Keywords:** functional MRI, language, semantic decisions, speech production, default network, words and objects

## Abstract

This fMRI study investigated the functional heterogeneity of the core nodes of the default mode network (DMN) during language processing. The core nodes of the DMN were defined as task-induced deactivations over multiple tasks in 94 healthy subjects. We used a factorial design that manipulated different tasks (semantic matching or speech production) and stimuli (familiar words and objects or unfamiliar stimuli), alternating with periods of fixation/rest. Our findings revealed several consistent effects in the DMN, namely less deactivations in the left inferior parietal lobule during semantic than perceptual matching in parallel with greater deactivations during semantic matching in anterior subdivisions of the posterior cingulate cortex (PCC) and the ventromedial prefrontal cortex (MPFC). This suggests that, when the brain is engaged in effortful semantic tasks, a part of the DMN in the left angular gyrus was less deactivated as five other nodes of the DMN were more deactivated. These five DMN areas, where deactivation was greater for semantic than perceptual matching, were further differentiated because deactivation was greater in (i) posterior ventral MPFC for speech production relative to semantic matching, (ii) posterior precuneus and PCC for perceptual processing relative to speech production, and (iii) right inferior parietal cortex for pictures of objects relative to written words during both naming and semantic decisions. Our results thus highlight that task difficulty alone cannot fully explain the functional variability in task-induced deactivations. Together these results emphasize that core nodes within the DMN are functionally heterogeneous and differentially sensitive to the type of language processing.

## Introduction

The “default network” or the “default mode network” (DMN) is a set of nodes that are more active in the absence of specific goal-directed tasks. This network has frequently been described as the reduction of activity in specific brain regions when subjects are engaged in effortful and focused external tasks (Shulman et al., 1997; Gusnard and Raichle, 2001; Mazoyer et al., 2001; Raichle et al., 2001), and it is easily visualized as a set of task-induced deactivations. These task-induced deactivations within the DMN are remarkably reliable (Shehzad et al., 2009) and consistent across different tasks, paradigms, subjects, and studies, see recent meta-analysis reviews (Buckner et al., 2008; Laird et al., 2009; Smith et al., 2009; Spreng et al., 2009; Biswal et al., 2010). In this paper, we aim to characterize the different (de)activation patterns in the core regions of the DMN while systematically varying language tasks and stimuli in a large group of 94 healthy subjects. More specifically, we tested how the deactivation level within the DMN varied with the demands on semantic, perceptual, and speech production processing.

Despite the huge amount of data regarding the DMN, there is still no consensus about its *function*. We do not know yet what core processes are sustained by the DMN in the healthy brain although, in general, the DMN seems critical in reasoning (Harrison et al., 2008) and in the interplay between self and external awareness (Boly et al., 2008). The neuroimaging literature has provided some valuable insights into the function of the DMN mainly along three perspectives. First, some studies have investigated the potential relationship between the DMN and other task-specific networks including for instance attention, memory, language (Binder et al., 1999, 2009; Mazoyer et al., 2001; Fox et al., 2005; Biswal et al., 2010; Kim, 2010; Mayer et al., 2010; Mennes et al., 2010; Seghier et al., 2010; Yang et al., 2010; Anderson et al., 2011; Sestieri et al., 2011; Wirth et al., 2011; Geranmayeh et al., 2012), and other higher cognitive systems such as auto-biographical memory, theory of mind, and prospection (see review in Buckner et al., 2008; Spreng et al., 2009). These studies have shown that the DMN overlaps (partially or completely) with these task-related networks and that the (de)activation level within the DMN can predict performance during different goal-directed tasks (e.g., Esposito et al., 2009; Anticevic et al., 2010). Second, instead of defining the DMN as one homogenous network, other studies have segregated the DMN into different functional components with high spatial definition (Laird et al., 2009; Seghier and Price, 2009; Andrews-Hanna et al., 2010; Kim, 2010; Mayer et al., 2010; Stawarczyk et al., 2011; Andrews-Hanna, 2012). For instance, different subdivisions have been shown to exist within the DMN nodes as has been shown for instance for the posterior cingulate cortex (PCC; Margulies et al., 2009; Leech et al., 2011). Third, other studies have characterized the differences in connectivity within the DMN or between the DMN and other networks (Uddin et al., 2009; Mennes et al., 2010; Jiao et al., 2011). For instance, it has been shown that changes in the inter-regional coupling with the DMN can predict behavioral performance during the processing of external information (Kelly et al., 2008; Sala-Llonch et al., 2012).

The aim of the current paper was to explore the response profiles in different parts of the DMN and language tasks that varied in access to semantics. The parallel between the default network and the semantic system has been suggested by Binder and colleagues (Binder et al., 1999, 2009; McKiernan et al., 2003, 2006) who proposed that task-unrelated thoughts that occurred during conscious passive states are essentially semantic because they depend on activation and manipulation of acquired knowledge about the world. Thus the engagement in effortful tasks reliably suppresses such task-unrelated thoughts (i.e., interrupting the stream of consciousness), suggesting a direct competition between exogenous and endogenous signals for attentional and executive resources. More specifically, Binder et al. (1999) observed that (i) subjects deactivated DMN regions during a perceptual decision task but not during a semantic decision task, and (ii) these regions were reliably deactivated during pseudoword processing but not during meaningful words and sentences (Binder et al., 2005; Humphries et al., 2007). Therefore, as discussed recently in Binder et al. (2009), Binder and Desai (2011), the default network regions assumed a critical role in concept retrieval and conceptual integration that are either activated in semantic tasks or deactivated during other active tasks.

However, this framework also recognized that the amplitude of the deactivation (iii) can be parametrically modulated by task difficulty (McKiernan et al., 2003) that required reallocation of processing resources when varying target discriminability and short term memory load, and (iv) was proportional to the level of the interruption of internal conscious processes during an auditory target detection task (McKiernan et al., 2006). Thus, the comparison between semantic and non-semantic processes might be confounded by differences in task demand. For that reason, our paradigm also manipulated different types of task demands that varied between demand on perceptual processing on meaningless stimuli or demand on semantics on familiar stimuli while varying task between semantic matching and speech production. Moreover, it was also critical here to take into account the functional heterogeneity of both systems. Indeed, different reports have shown reliable functional parcellations of both the semantic system (Postler et al., 2003; Binder et al., 2009; Seghier and Price, 2009; Wu et al., 2009; Sharp et al., 2010; Kim et al., 2011) and the DMN (Laird et al., 2009; Seghier and Price, 2009; Andrews-Hanna et al., 2010; Kim, 2010; Mayer et al., 2010; Stawarczyk et al., 2011; Andrews-Hanna, 2012), although the exact relationship between the two systems at such high spatial definition remains unclear (e.g., Laird et al., 2009; Andrews-Hanna et al., 2010; Seghier et al., 2010; Wirth et al., 2011).

In our previous work, we have shown that the deactivated patterns in the DMN during semantic matching on words can be dissociated into two components (Seghier and Price, 2009), one that was deactivated “similarly” between perceptual and semantic matching and a second component that was deactivated “differently” between semantic and perceptual matching (e.g., see Figure 4 in Seghier and Price, 2009). We also identified a reliable overlap between the DMN and the semantic system at the level of the left angular gyrus (Seghier et al., 2010). Here we aim to investigate the following issues on the same cohort of 94 healthy subjects. First, we characterize the task-dependent deactivations for semantic and non-semantic conditions in the core regions of the default network that included bilateral inferior parietal lobule (IPL), precuneus and PCC, and medial prefrontal cortex (MPFC). We predict that the amplitude of deactivations would vary with task and stimulus (Harrison et al., 2011), for instance varying between semantic versus perceptual matching or between speech production and matching tasks. Second, we predict that posterior and anterior regions of the DMN would show different dependencies with task demand (Lin et al., 2011). Third, by mapping these effects at the voxel level, functional subdivisions can be segregated within each node of the DMN (Andrews-Hanna, 2012). Last but not least, we also explicitly tested whether other demographic or behavioral factors may explain some of the functional heterogeneity within the DMN.

## Materials and Methods

### Subjects

The data from 94 subjects (aged 30.8 ± 15.8 years, 50 females, 44 males) were included in our group analyses. According to the Edinburgh handedness questionnaire (Oldfield, 1971), 54 were right-handed and 40 were either left-handed or ambidextrous. All subjects were native English speakers, had normal or corrected-to-normal vision, and had no history of neurological or psychiatric disorders. The inclusion of a large heterogeneous sample of subjects who differed in their handedness, age, and gender allows our findings to be generalized across different populations as well as giving us the opportunity to explicitly investigate the influence of these demographic variables on brain activity in different regions. More details about the subjects can be found in Seghier et al. (2010).

The study was approved by the National Hospital for Neurology and Institute of Neurology Joint Ethics Committee.

### Experimental design

**Figure 1:**
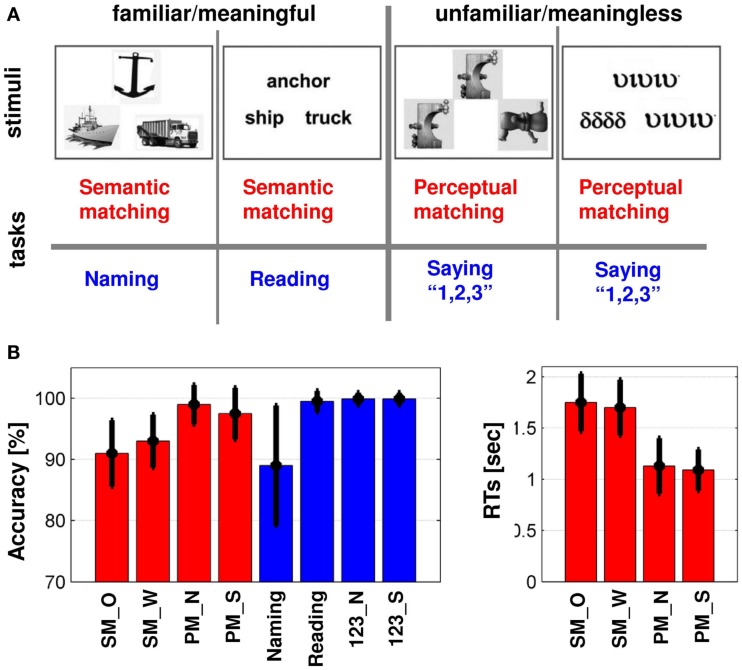
**(A)** Illustrates the eight conditions in our fMRI sessions, including matching tasks (red) and production tasks (blue). The eight conditions alternated with periods of fixation where subjects were asked to fixate on a central cross and rest. **(B)** Summary of in-scanner behavioral responses of all conditions: mean (±standard deviation) of accuracy [in (%)] and reaction times [RTs in (s)] during all conditions over our 94 subjects. All subjects performed the tasks with high accuracy. SM_O, semantic matching on objects; SM_W, semantic matching on words; PM_N, perceptual matching on non-objects; PM_S, perceptual matching on symbols; 123_N, say “1,2,3” to non-objects; 123_S, say “1,2,3” to symbols.

Our participants were engaged in eight goal-directed tasks as well as fixation (Figure 1A). The semantic network was identified where activation was observed for two semantic decision tasks on (1) familiar pictures of objects and (2) their written names relative to two perceptual decision tasks on (3) unfamiliar pictures of meaningless non-objects and (4) unfamiliar Greek letter strings (e.g., Vandenberghe et al., 1996; Josse et al., 2008). There were also four speech production tasks that involved (5) naming pictures of familiar objects; (6) reading aloud written object names, (7) saying “1,2,3” to unfamiliar pictures of meaningless non-objects, and (8) saying “1,2,3” to unfamiliar Greek letter strings; see (Josse et al., 2008, 2009; Seghier et al., 2010; Seghier and Price, 2011) for further details. The DMN was defined as task-induced deactivations for all eight tasks relative to fixation (e.g., Shulman et al., 1997; Laird et al., 2009), see below for more details.

### Experimental procedures

In one session, the participants made semantic and perceptual decisions (i.e., the four conditions shown in red in Figure 1A), interleaved with blocks of fixation. This session was repeated with a different set of stimuli and a different order of conditions. In another session, the participants performed the speech production tasks (i.e., the four conditions shown in blue in Figure 1A), interleaved with blocks of fixation. This session was also repeated with a different set of stimuli and a different order of conditions. This yielded a total of four separate scanning runs or sessions for each participant in the same fMRI experiment. The order of conditions was thus counter-balanced within and across session. Each session consisted of 24 blocks of stimuli of the same type/condition with an additional 12 blocks of fixation that were presented every two stimulus blocks. Each stimulus block lasted 18 s and consisted of four trials during which three stimuli were simultaneously presented on the screen for 4.32 s, followed by 180 ms of fixation. Every two stimulus blocks, fixation continued for 14.4 s.

All stimuli were presented in triads with one item (picture or letter string) above and two items below in the same format as the item above (Figure 1A). During semantic and perceptual decisions, the item above acted as a target that was semantically or physically related to one of the items below. Specifically, subjects were asked to indicate by a finger press response whether the target stimulus was semantically related or perceptually identical to the stimulus on the lower-left or lower-right. In the speech production conditions, there was no semantic or perceptual relationship between any of the three items, and subjects were asked to name each of the three objects in the pictures aloud, read aloud each of the three words, or say “1,2,3” while looking at each of the three pictures of meaningless non-objects or the three strings of greek letters. Prior to each stimulus block, a brief instruction was presented on the screen for 3.6 s to indicate what sort of response would be necessary. Stimulus presentation in the scanner was via a video projector, a front-projection screen, and a system of mirrors fastened to the MRI head coil. Additional details about the stimulus selection can be found in Seghier et al. (2010), Seghier and Price (2011). Responses during the matching task were recorded using a button box held under one hand throughout the experiment. As handedness varied between our participants, the hand used during the matching conditions was counter-balanced across participants. More specifically, 57 subjects responded with the right hand by indicating the lower-left stimulus with their first finger and the lower-right stimulus with their middle finger. Likewise, 37 subjects responded with their left hand by indicating the lower-left stimulus with their middle finger and the lower-right stimulus with their first finger. The hand of response was not determined by the hand the subject used to write with. Approximately, half the left handers responded with their right hand and the other half with their left hand. Likewise half the adult right handers responded with their left hand. Critically, however, the same hand of response was used in the semantic and perceptual conditions during both sessions. Therefore differences in left and right hand responders were removed when perceptual matching activation was subtracted from semantic matching activation (for more details, see Seghier et al., 2011).

### In-scanner behavioral responses and task difficulty

We measured task difficulty in terms of processing time (RTs) and errors. For instance, previous studies have shown that object naming is more difficult (time consuming) than word reading (Fraisse, 1969; Potter and Faulconer, 1975). Our data also showed that semantic matching was more difficult (slow RTs and more errors) than perceptual matching (as illustrated in Figure 1B). More specifically, the range of RTs varied from 0.95 to 2.6 s for semantic decisions on familiar stimuli and from 0.68 to 2.24 s for perceptual decisions on unfamiliar stimuli. Over our 94 subjects (Figure 1B), the differences in in-scanner behavioral responses were as following: (i) RTs were greater (slower) for semantic matching than perceptual matching irrespective of stimuli (*p* < 0.001), (ii) accuracy was lower during semantic matching than perceptual matching irrespective of stimuli (*p* < 0.001), (iii) accuracy was lower during semantic matching on pictures than words (*p* = 0.01), (vi) accuracy was lower during naming than reading or saying “1,2,3” (*p* < 0.001), and (v) accuracy during naming was lower than all the other matching conditions (*p* < 0.001) except semantic matching on objects where the effect was only a trend (*p* = 0.07). Accordingly, task demand varied considerably across our eight conditions, with task difficulty in terms of errors being high during naming and semantic matching, intermediate for perceptual matching, and low during reading and saying “1,2,3” to unfamiliar stimuli.

### MRI acquisition

Experiments were performed on a 1.5 T Siemens system (Siemens Medical Systems, Erlangen, Germany). Functional imaging consisted of an EPI GRE sequence (repetition time/echo time/flip angle = 3600/50 ms/90°, field of view = 192 mm, matrix = 64 × 64, 40 axial slices, 2 mm thick with 1 mm gap). Functional scanning was always preceded by 14.4 s of dummy scans to insure steady-state tissue magnetization. To ensure all the components of the hemodynamic response are effectively sampled, we used a distributed sampling by simply introducing a mismatch between stimulus presentation and repetition time (Veltman et al., 2002). To avoid ghost-EPI artifacts, image reconstruction was based on a generalized algorithm (i.e., trajectory-based reconstruction after calibrating a trajectory scan during a gel-phantom experiment). Anatomical T1-weighted images were acquired using a three-dimensional modified driven equilibrium Fourier transform sequence (176 sagittal slices, image matrix = 256 × 224, final resolution = 1 mm^3^, repetition time/echo time/inversion time = 12.24/3.56/530 ms).

### fMRI data analysis

Data processing and statistical analyses were performed with the Statistical Parametric Mapping SPM5 software package (Wellcome Trust Centre for Neuroimaging, London UK, http://www.fil.ion.ucl.ac.uk/spm/). All functional volumes were spatially realigned, un-warped, normalized to MNI space using the unified normalization-segmentation procedure of SPM5, and smoothed with an isotropic 6-mm full-width at half-maximum Gaussian kernel, with resulting voxels size of 2 mm × 2 mm × 2 mm. The normalization to the MNI space was performed by first coregistering the anatomical T1 image to the mean EPI image that was generated during the realignment step, then the unified segmentation was applied to the coregistered anatomical image using the default parameters in SPM5 to estimate the normalization parameters that encode the transformation from the native to MNI space, and finally the normalization parameters were subsequently applied to all realigned EPI images. Time-series from each voxel were high-pass filtered (1/128 Hz cut-off) to remove low frequency noise and signal drift. The pre-processed functional volumes of the four fMRI sessions of each subject were then submitted to a fixed-effects analysis, using the general linear model at each voxel. Each stimulus onset was modeled as an event using condition-specific “stick-functions” having a duration of 4.32 s per trial and a stimulus onset interval of 4.5 s. Correct responses for each condition, instructions, and errors were modeled separately (i.e., as separate regressors) in the design matrix. These were convolved with a canonical hemodynamic response function thus providing regressors for the linear model. As in standard SPM procedures, the design matrix also included four (constant) regressors to model the average signal in each session. The contrast images for each of the eight conditions (correct trials only) compared to fixation were then entered into a second-level analysis (i.e., random-effects analysis) to enable inferences at the group level. Our second-level analyses systematically explored the direction (activation or deactivation) and amplitude of the signal change (see similar rationale in Box 2 of Gusnard and Raichle, 2001). The main group effects of interest are reported at *p* < 0.05 FWE-corrected for multiple comparisons across the whole brain with a minimum cluster size of 10 voxels in the group analysis. Finally, we compared the significant clusters to the probabilistic cytoarchitectonic maps within the Anatomy Toolbox (v1.8, Eickhoff et al., 2005), although such maps do not cover MPFC and the whole PCC regions.

### The default mode network

**Figure 2:**
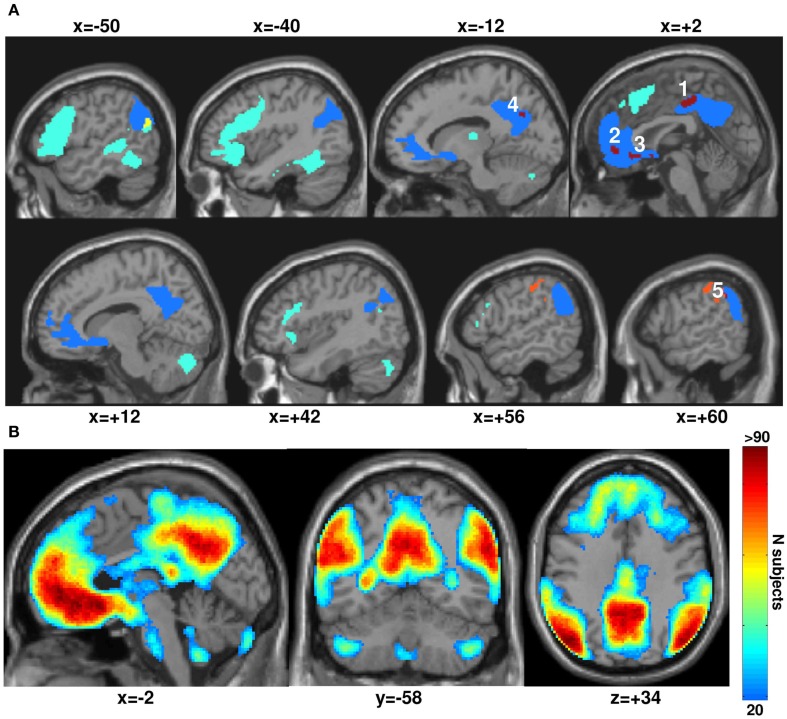
**(A)** Main effect from the group analysis (at *p* < 0.05 FWE-corrected) over our 94 subjects. In blue: the default mode network (DMN) showing the task-induced deactivations for all conditions relative to fixation. In green: semantic matching relative to perceptual matching (semantic system) that is outside the DMN. In yellow: the semantic system within the DMN (at the level of the left angular gyrus). In orange: perceptual matching relative to semantic matching outside the DMN. In dark red: perceptual matching relative to semantic matching within the DMN. For more consistency, our five regions of interest (in dark red) are labeled with the same numbers as in Figure 4 and Table 1. All significant voxels are projected on an individual T1-weighted image on sagittal views varying from *x* = −50 to *x* = +60 mm. **(B)** Overlap between the individual task-induced deactivations (DMN defined in each subject at a low threshold of *p* < 0.05 uncorrected). These percent of overlap maps measure how many subjects are deactivating each voxel of the DMN at a given individual threshold. Voxels in red are highly consistent across our 94 subjects.

The DMN was identified as the main average effect of fixation relative to all stimuli irrespective of task or modality (i.e., deactivations at *p* < 0.05 FWE-corrected), see Figure 2. Although many fMRI studies segregated the DMN from resting-state activity by seed-based correlations analysis or data-driven approaches such as independent component analysis (see review in Cole et al., 2010; Van Dijk et al., 2010), we used the alternative approach as in many other studies that defined the default network as a set of task-induced deactivations relative to a fixation (rest) condition (see meta-analysis review in Laird et al., 2009) and found robust and reliable results (Shulman et al., 1997; Mazoyer et al., 2001; Raichle et al., 2001). Indeed, our results showed remarkable consistency of the DMN across our 94 subjects as illustrated in Figure 2B using the percent of overlap maps that measure how many subjects are deactivating each voxel at a given threshold (for a similar procedure, see Seghier et al., 2008).

It is worth noting that the definition of the DMN was restricted here to its core regions where task-related deactivation was most significant (*p* < 0.05 FWE-corrected over the whole brain): IPL, MPFC, precuneus, and PCC (for review, see Buckner et al., 2008). As a consequence of this strict statistical threshold, other regions associated with the DMN, including middle temporal, cerebellar, or hippocampal areas were excluded even though bilateral hippocampal regions (at *x* = 28 *y* = −16 *z* = −18; *x* = −24 *y* = −20 *z* = −18) and lateral temporal areas (*x* = 58 *y* = −4 *z* = −18; *x* = −56 *y* = −4 *z* = −22) were deactivated but with less consistency across subjects (Figure 2B). Somatosensory cortices where deactivation has previously been reported for production tasks, such as reading and naming (e.g., Guenther et al., 2006; Dhanjal et al., 2008; Ventura et al., 2009; Behroozmand and Larson, 2011; Houde and Nagarajan, 2011) were also excluded.

Our finding that deactivations in the hippocampal and middle temporal regions were less robust and less consistent across subjects is in line with previous studies that identified the DMN using task-induced deactivations (see meta-analysis in Laird et al., 2009) rather than with resting-state functional connectivity (see Discussion in Buckner et al., 2008). Moreover, a recent connectivity analysis that assessed the causal interactions between the different nodes of the DMN showed that the regions we identified with the DMN have the highest activity levels as measured by the power of low frequency BOLD fluctuations (Jiao et al., 2011). They also have very strong positive intrinsic correlations among themselves compared to any other DMN regions (see Figure 8 in Buckner et al., 2008). In summary, the core DMN regions considered here were those that have been shown to be most consistent and robust in previous reports (see Damoiseaux et al., 2006; Shehzad et al., 2009; Smith et al., 2009).

### The semantic network

The semantic system was identified by comparing semantic matching on familiar words and objects to perceptual matching on unfamiliar Greek symbols and non-objects (Figure 2A). This contrast identified strongly left-lateralized activation in parietal, temporal, and frontal regions with right-lateralized activation in the cerebellum (cf. Seghier et al., 2010).

### Effects of interest

Our factorial experimental design allowed the influence of different factors on the (de)activation level in different parts of the DMN to be assessed. We focus here on two main analyses within the voxels of interest of Figure 2A: (1) identify regions of interest that were strongly deactivated during semantic matching using second-level group analyses, and (2) test whether the effects identified in (1) were explained/caused by the heterogeneity of our group of healthy subjects, including effects that may vary with age, gender, handedness, or language lateralization. The details of such analyses are described below.

For the interpretation of the direction of the effects in each node of the DMN, we conventionally described each effect as being less or more deactivated relative to a particular baseline (fixation or a control condition). This would ensure that our interpretations follow the same rationale as in previous fMRI studies that investigated task-induced deactivations. For instance, difficult tasks were previously portrayed as yielding greater deactivation than easy/simple tasks, although a more orthodox interpretation would report the same effect as a stronger activation for easy versus difficult tasks. The same principle applies for semantic processing where semantic tasks yielded less deactivation than perceptual tasks in some of the DMN regions. We believe this would make the interpretation of the direction of the effects easier because we are essentially dealing with differences in deactivations within the DMN. To clarify the direction of our effects, we report the conditions contrasted for each finding (see Results below).

### Semantic versus perceptual matching

We first contrasted semantic matching on familiar stimuli to perceptual matching on unfamiliar stimuli within the core regions of the DMN. Both tasks involved strong demand on perceptual processing, attention to the visual stimuli, and making decisions, but semantic matching required explicit semantic associations to be made. We previously used this contrast to visualize how the DMN overlapped with the semantic system (Seghier et al., 2010), shown in yellow in Figure 2A. Here we predicted greater deactivations in the core DMN regions for semantic than perceptual matching because semantic matching was more difficult (slower RTs and more errors) than perceptual matching (Figure 1B), and task demand/difficulty was expected to impact upon the deactivation signal within the DMN (McKiernan et al., 2003, 2006; Singh and Fawcett, 2008; Lin et al., 2011). Greater deactivation for semantic than perceptual matching should therefore reveal DMN regions that were more suppressed when the external task-related processes required extra semantic demands (illustrated in dark red in Figure 2A). The response profiles of the regions of interest identified above were then investigated by comparing perceptual matching to saying “1,2,3” and words to objects, as detailed below.

### Production versus perceptual matching

The perceptual matching task required attention to unfamiliar visual stimuli whereas saying “1,2,3” was unrelated to the visual stimuli but required attention to the articulatory output. As, both perceptual matching with saying “1,2,3” were in response to the same stimuli, the influence of external semantic, or conceptual processing was minimized (Figure 1A). We also tested for the effect of speech production during naming and reading, relative to semantic decisions on the same stimuli.

### Word versus object stimuli

For pictures of objects, visual input is linked first to semantics and then to speech production but when the stimuli are written words, visual inputs (i.e., orthography) can also be linked directly to phonology without access to semantics. Written words can also access semantics from phonology. This means that, during semantic decisions, written words may activate phonology more than pictures, whereas during speech production, pictures may activate semantics more than written words (Glaser and Glaser, 1989).

### Influence of the degree of language lateralization

Importantly, because of the well-known relationship between language laterality and handedness (e.g., Pujol et al., 1999; Knecht et al., 2000; Szaflarski et al., 2002), we aimed to test whether inter-individual variability in deactivation level in DMN can be explained by differences in language lateralization (Liu et al., 2009; Swanson et al., 2011; Tian et al., 2011). To express the relative difference in the involvement of the left versus right hemisphere regions, we computed the language laterality index for each subject (Seghier, 2008) using Nagata et al.’s (2001) approach that is independent of the statistical threshold. In brief, this procedure calculates the number of left and right hemisphere voxels activated for a given contrast, at a range of different statistical thresholds. Non-linear regression of the shape of the curve, describing the relationship between the number of voxels and the statistical threshold, provides a constant term that is used to compute a normalized difference between left and right hemisphere activity (Nagata et al., 2001). A positive index (toward +1) indicates left hemisphere dominance, whereas a negative index (toward −1) indicates right hemisphere dominance. Here we computed language laterality indices from the contrast of object naming versus saying “1,2,3” using regions of interest covering the whole frontal lobe and. Laterality indices during object naming are reliable measures for language lateralization at the individual level (Petrovich Brennan et al., 2007). In our group of 94 healthy subjects, the degree of lateralization during object naming in frontal regions varied across subjects, with 23 subjects having negative language laterality indices and 71 subjects having positive language laterality indices (mean laterality indices = 0.17 ± 0.28). These laterality indices were subsequently included as a covariate of interest in second-level regression analyses (across subjects) using either the contrast images of the semantic matching or perceptual matching as the dependent variable.

### Influence of demographic variables and RTs

We also evaluated the influence of age, gender, handedness, and RTs on deactivations across our 94 subjects. Previous work have shown that demographic variables may impact on the DMN and other resting-state networks (Wang et al., 2008; Biswal et al., 2010; Grady et al., 2010; Sambataro et al., 2010; Allen et al., 2011; Dong et al., 2012; Filippi et al., 2012; Spreng and Schacter, 2012); however there is no consensus in this literature regarding the exact impact of such variables on the functional properties of each node of the DMN. Therefore it was not possible to generate specific predictions although it remained plausible that these factors influence the deactivation level in one or more of the DMN regions. To do that, we carried out (i) a second-level multi-regression analysis using age and RTs as covariates of interest for each condition relative to fixation, and (ii) a second-level factorial design with gender, handedness, and task as factors of interest. The significant effects of interest were limited to the core DMN regions.

Note that our paradigm and analysis were not optimized for testing the influence of task difficulty because in-scanner RTs (i) were not collected during the two speech production sessions, (ii) were not explicitly included as parametric modulations in the first-level analysis at the individual subject level, and (iii) subjective difficulty (i.e., how subjects rated each task in terms of difficulty) was not recorded. Accordingly, the effect of RTs was assessed here for the matching conditions only using a between-subject second-level analysis that ignored the influence of inter-trial variability in RTs. Thus, within each of the matching conditions, our analysis aimed to look at the impact of inter-subject differences in RTs upon the deactivation level of each voxel of the DMN.

## Results

### Greater deactivations for semantic than perceptual matching

**Figure 3:**
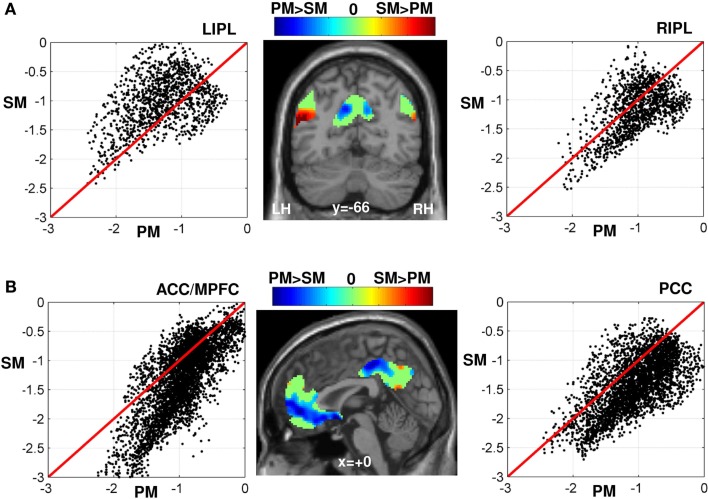
**(A)** Scatter plot of all voxels within the LIPL (left) and RIPL (right) according to the amplitude of the (de)activations during semantic (SM) versus perceptual matching (PM). The amplitude of the (de)activation was measured by the effect size or the weighted-beta values of each condition in the general linear model. Voxels above the red line of each scatter plot were less deactivated during SM than PM and vice-versa. The projection of all voxels of the DMN on an individual T1-weighted image (middle panel) is displayed in blue-to-green-to-red color coding (on a coronal view at *y* = −66 mm) according to the difference in (de)activation amplitude of SM minus PM: less deactivations (in red) during SM than PM (mainly located in the left inferior parietal cortex, cf. reported and discussed in our previous work; Seghier et al., 2010), greater deactivations (in blue) during SM than PM, and in green similar deactivation level during both SM and PM. **(B)** idem as **(A)** in the midline regions PCC and MPFC (projected on a sagittal view at *x* = 0). Voxels shown in blue (middle panel) are strongly deactivated during SM than PM (limited to the midline regions of the DMN). For illustration purpose, all voxels with a weak difference in effect size between SM and PM are set to zero in the color-coded scale (green color). SM, semantic matching; PM, perceptual matching; LIPL, left inferior parietal lobule; RIPL, right inferior parietal lobule; ACC/MPFC, anterior cingulate cortex/medial prefrontal cortex; PCC, precuneus and posterior cingulate cortex; LH, left hemisphere; RH, right hemisphere. The effects that were significant at *p* < 0.05 FWE-corrected are visualized in Figures 2A and 4.

**Figure 4:**
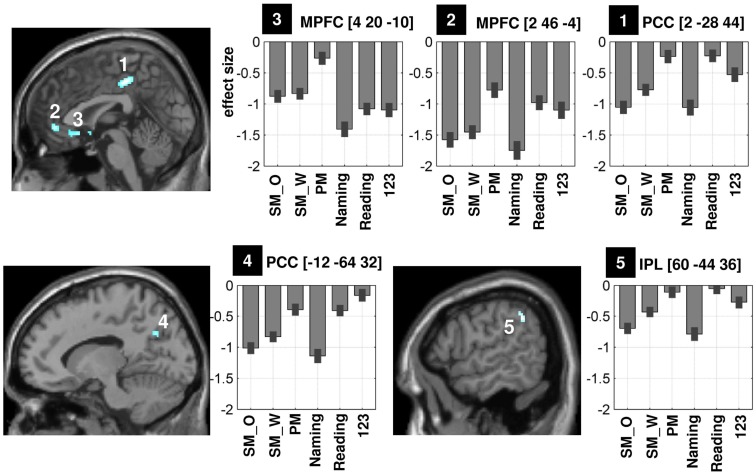
**Illustration of the mean effect size of five regions that showed greater deactivations during semantic matching than perceptual matching (at *p* < 0.05 FWE-corrected, see Table 1)**. There were two significant clusters in PCC (labeled “1” and “4”), two clusters in ventral MPFC (labeled “2” and “3”), and one cluster in RIPL (labeled “5”) on the sagittal view. Because differences between non-objects and Greek symbols were not significant, the effect sizes (in the bar graphs) for the unfamiliar stimuli were averaged to simplify the illustration. Coordinates are shown on top of each bar graph. PCC, precuneus and posterior cingulate cortex; MPFC, medial prefrontal cortex; RIPL, right inferior parietal lobule; SM_O, semantic matching on objects; SM_W, semantic matching on words; PM, perceptual matching on unfamiliar stimuli; 123, say “1,2,3” to unfamiliar stimuli.

Figure 3 illustrates the difference in deactivation amplitude in each voxel of the DMN during semantic matching versus perceptual matching. The majority of voxels that showed greater deactivations during semantic than perceptual matching were located in the midline DMN regions (voxels in dark blue in Figure 3B). More specifically, the statistical comparison (at *p* < 0.05 FWE-corrected) between perceptual matching and semantic matching indentified five clusters: (i) in anterior PCC (*x* = 2 *y* = −28 *z* = 44, *z*-score = 6.4), (ii) in posterior PCC (*x* = −12 *y* = −64 *z* = 32, *z*-score = 5.6), (iii) in anterior-ventral MPFC (*x* = 2 *y* = 46 *z* = −4, *z*-score = 5.9), (iv) in posterior-ventral MPFC (*x* = 4 *y* = 20 *z* = −10, *z*-score = 5.7), and (v) in right IPL (*x* = 60 *y* = −44 *z* = 36, *z*-score = 5.2), see Table 1. Comparing the location of our regions of interest to the cytoarchitectonic maps revealed that anterior PCC was bounded dorsally by area SPL-5M (Scheperjans et al., 2008), posterior PCC was more likely to be located in area SPL-7A, and the right IPL was located anteriorly to area PGa and mainly included area IPC-PFm (Caspers et al., 2008). The deactivation profiles of these five DMN regions are illustrated in Figure 4. Below, we considered how deactivation varied with task and stimuli in each region.

**Table 1:** **The differential effect of language processing in the five regions of interest**.

Contrast	(1) aPCC	(2) avMPFC	(3) pvMPFC	(4) pPCC	(5) RIPL
	2 −28 44	2 46 −4	4 20 −10	−12 −64 32	60 −44 36
PM > SM	**6.4**	**5.9**	**5.7**	**5.6**	**5.2**
123 > PM	n.s.	n.s.	n.s.	**4.8^a^**	n.s.
Matching > speech	n.s.	n.s.	**6.6^b^**	n.s.	n.s.
PM > 123	3.2	n.s.	**6.8^b^**	n.s.	n.s.
SM_W > SM_O	3.4	n.s.	n.s.	n.s.	**5.4^c^**

*^a^The global peak for this contrast was located at (−2 −72 38, *z*-score = 5.4)*.

*^b^The global peak of this contrast was located at (6 16 −8, *z*-score = 7.7)*.

*^c^The global peak of this contrast was located at (54 −52 48, *z*-score = 6.0)*.

*In bold: effects significant at *p* < 0.05 FWE-corrected*.

*n.s., Not significant at *p* < 0.001 uncorrected within a 4-mm-radius sphere centered at each peak*.

**Figure A1:**
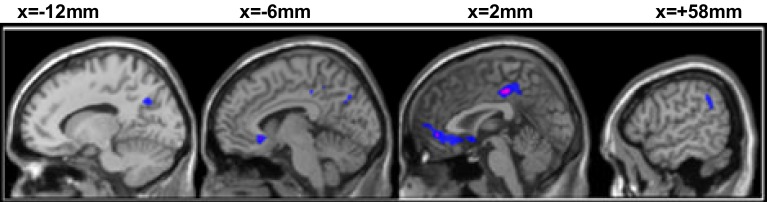
**Greater deactivations within the DMN for semantic than perceptual matching (contrast: PM > SM), at *p* < 0.05 FWE-corrected (magenta) and at *p* < 0.001 uncorrected (blue)**. This second-level group analysis was carried out on 71 out of 94 subjects who had positive language laterality indices (LIs assessed within the frontal lobe during the object naming task, see Materials and Methods for more details). The peak coordinates of the five regions of interest from this new analysis were: aPCC (4 −28 44, *z* = 5.9); avMPFC (2 46 −4, *z* = 5.2); pvMPFC (−4 24 −8, *z* = 4.9); pPCC (−12 −64 34, *z* = 4.7); RIPL (60 −42 40, *z* = 4.6). This analysis replicated the same effects as those identified over the whole group of 94 subjects (main text). More specifically, and in line with Table 1, perceptual matching on unfamiliar stimuli showed greater deactivations than saying “1,2,3” in pPCC (peak at −2 −72 38, *z* = 4.6) whereas the reverse was significant in pvMPFC (global peak at 6 16 −10, *z* = 7.0). Last but not least, semantic matching on objects showed greater deactivations than semantic matching on words in RIPL (peak at 52 −54 48, *z* = 5.3); for more details, see Table 1 of the main text.

*The significance in *z*-scores for five different statistical contrasts in the five regions of interest identified by the contrast PM > SM. Contrasts are described in the conventional way of an activation condition relative to a control condition (e.g., PM > SM means greater deactivation in SM). SM, semantic matching on familiar stimuli; PM, perceptual matching on unfamiliar stimuli; 123, say “1,2,3” to unfamiliar stimuli; SM_O, semantic matching on objects; SM_W, semantic matching on words. Regions are listed in the same labels as in Figure 4. These effects remain significant after excluding the 23 subjects with negative language laterality indices (for more details, see Figure A1 in Appendix)*.

### Functional specialization in the five DMN regions

First, the demands on perceptual processing (Table 1), as measured by greater deactivation for perceptual matching than saying “1,2,3” on unfamiliar stimuli, resulted in significantly more deactivation in posterior PCC than in any of the other regions (i.e., significant region-by-condition interaction: *F* = 7.8, *p* < 0.001; all paired *t*-tests for anterior PCC versus other regions were significant at *p* < 0.05 uncorrected). This suggests that only posterior PCC was strongly influenced by the demands on perceptual processing and visual attention (see bottom-left panel of Figure 4, fifth column of Table 1).

Second, the demands on speech production, as measured by saying “1,2,3” relative to perceptual matching on unfamiliar stimuli, resulted in stronger deactivation in posterior-ventral MPFC than in any of the other regions (i.e., all paired *t*-tests between posterior-ventral MPFC and the other regions were significant at *p* < 0.005 uncorrected). The effect of speech production on posterior-ventral MPFC deactivation was also significant when the stimuli were both familiar and unfamiliar (see top-left panel of Figure 4, fourth column of Table 1). Third, the demands on picture processing, as measured by greater deactivations for semantic matching on objects than words, were stronger in an anterior cluster of the right IPL (see bottom-right panel of Figure 4, sixth column of Table 1) than all regions (*p* < 0.05 in each paired *t*-test) except the anterior PCC (paired *t*-test not significant at *p* < 0.05). Fourth, deactivation in anterior PCC and anterior-ventral MPFC were best explained by the demands on semantics because they were uninfluenced by the demands on perceptual processing or speech production (see second and third columns of Table 1; top-right and top-middle panels of Figure 4).

### Other factors influencing deactivation in the five DMN regions

For each of the five DMN clusters, we then investigated whether deactivations correlated with age, gender, language laterality, or task difficulty (RTs and errors) across our 94 subjects. In posterior PCC, deactivation was greater for (i) females than males during perceptual matching at (*x* = −14 *y* = −64 *z* = 34, *z*-score = 3.2; *p* < 0.001 uncorrected); and (ii) as error rate increased in all conditions (i.e., the correlation between error rate of Figure 1B and the response profile of Figure 4 was very significant: *r* = 0.97, *p* < 0.001).

There were no other significant effects in the five regions of interest even when the statistical threshold was *p* < 0.001 uncorrected. The absence of significant correlations within the five regions of interest was unlikely to be due to insufficient sensitivity to detect these effects in our large sample of subjects. This is because, in other parts of the DMN, we found a highly significant effect of age in posterior-ventral PCC during semantic matching (*x* = −18 *y* = −68 *z* = 20, *z*-score = 5.2; at *p* < 0.05 FWE-corrected), with greater deactivations for younger than older subjects. At a lower threshold of *p* < 0.001 uncorrected, we also found (i) correlations with language laterality in right IPL during semantic matching at (*x* = 54 *y* = −54 *z* = 28, *z*-score = 4.2), with greater deactivation in subjects with stronger left-lateralized language, (ii) females tended to show stronger deactivations than males over all tasks in the left IPL (*x* = −44 *y* = −68 *z* = 24, *z*-score = 4.0) and posterior MPFC (*x* = −2 *y* = 58 *z* = −4, *z*-score = 3.8), and (iii) correlations with RTs in the right IPL (at *x* = 48 *y* = −50 *z* = 42, *z*-score = 3.6) during semantic matching on objects, with greater deactivation in subjects with faster RTs.

## Discussion

**Figure 5:**
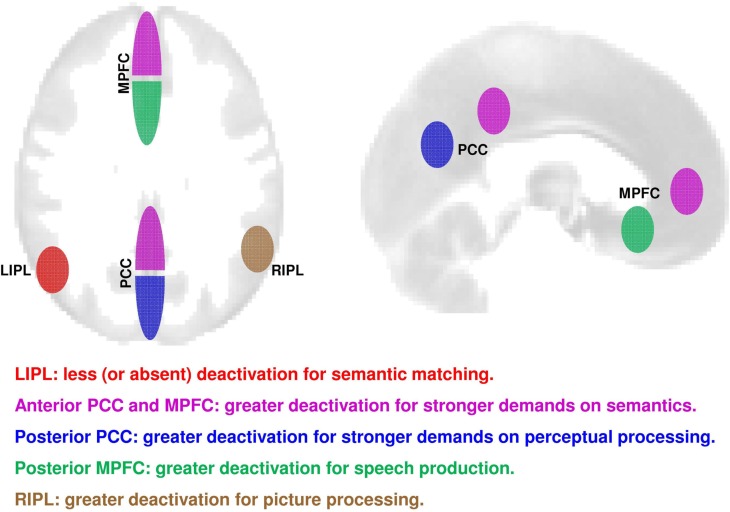
**A schematic summary on axial (left) and sagittal (right) views of the consistent effects over all our 94 subjects in the different core regions of the DMN**. In red: less deactivation during semantic matching (contrast = SM > PM). In magenta: strongly deactivated during semantic matching than perceptual matching (contrast = PM > SM); in blue: deactivation related to task demand for perceptual processing (contrast = say “123” > PM). In green: greater deactivation during speech production over both familiar and unfamiliar stimuli (contrast = matching > production). In brown = greater deactivation for pictures of objects than to written words (contrast = words > pictures). PCC, precuneus and posterior cingulate cortex; MPFC, medial prefrontal cortex; LIPL, left inferior parietal lobule; RIPL, right inferior parietal lobule.

This study investigated how deactivation within core regions of the DMN was influenced by language tasks and stimuli. Our findings show significantly different patterns of deactivation in core DMN regions during perceptual, semantic, and speech production tasks (see schematic illustration in Figure 5). First, we identified five subdivisions where deactivation was greater for semantic matching than perceptual matching; a profile which reversed from that observed in the left angular gyrus (Seghier et al., 2010) where deactivation was less for semantic than perceptual matching. Second, within the five regions showing greater deactivations for semantic than perceptual matching, we dissociated four different response profiles. Specifically, deactivation was stronger during (i) speech production in posterior-ventral MPFC, (ii) perceptual processing in posterior PCC, (iii) semantic processing in anterior PCC, right IPL, and anterior-ventral MPFC, and (iv) picture processing in right IPL. These effects were highly consistent across our heterogeneous group of healthy subjects as deactivation did not significantly correlate with age, gender, handedness, language laterality, or RTs. Below, we discuss how these findings compare with previous results about the involvement of the DMN regions in task-related processes (Harrison et al., 2008; Esposito et al., 2009; Laird et al., 2009; Seghier and Price, 2009; Smith et al., 2009; Spreng et al., 2009; Uddin et al., 2009; Anticevic et al., 2010; Kim, 2010; Mayer et al., 2010; Mennes et al., 2010; Stawarczyk et al., 2011; Andrews-Hanna, 2012; Seghier, 2012).

Previous studies have investigated the functional responses and connectivity of language regions during rest (Koyama et al., 2010, 2011; Zhao et al., 2011; Tomasi and Volkow, 2012), namely by characterizing fMRI signal and connectivity during rest in a set of predefined language regions. Here we took the opposite approach by looking at how deactivation during rest/fixation was influenced by language tasks and stimuli in a set of core DMN regions. Other studies (e.g., Laird et al., 2009; Kim, 2010) have attributed some specific roles to each node of the DMN on the basis of other functional studies reporting activation in the same areas during specific tasks. In our study, both the DMN and language activations were assessed in the same experimental setting and in the same group of healthy subjects, which allowed direct comparisons between the two to be made using standard univariate whole brain subtraction analyses.

### Impact of semantic processing on the DMN

Although previous work predicted the DMN would be less (or not at all) deactivated during semantic processing (e.g., Binder et al., 2009; Binder and Desai, 2011), we found that large parts of the DMN showed comparable deactivations during semantic and perceptual matching (voxels in green in Figure 3). For instance, Binder and colleagues showed that semantic decision tasks did not deactivate the DMN whereas a perceptual task with no semantic content strongly deactivated the DMN (see Figure 8 of Binder et al., 2009). In our study, however, this prediction was replicated only in the left IPL, perhaps because our semantic task did not involve particularly challenging semantic manipulations. Alternatively, deactivation may be influenced by two competing factors with opposite effects: semantic demands that reduce deactivation in the DMN versus task demands (difficulty) that increases deactivation in the DMN. The sum effect may vary across the core regions of the DMN thereby explaining why reduced deactivation during semantic tasks does not always overlap with the DMN (Figure 3). For example, in the right IPL region of the DMN, there is a weak effect of semantics, consequently, the DMN is more symmetrical than the semantic system; for a discussion, see Seghier et al. (2010).

Interestingly, the impact of semantic processing seems to play contrasting roles on different parts of the DMN. More specifically, there was a dissociation in semantics between left IPL and two anterior parts of the PCC and MPFC (Figures 3 and 4). In left IPL, in a cluster mainly located in area PGp (Caspers et al., 2008), deactivation was less during semantic than perceptual matching. This was documented in our previous report (cf. Seghier et al., 2010) and fits well with prior reports that the left IPL, particularly the angular gyrus, is involved in semantic processing (for recent review, see Seghier, 2012). For instance, in their meta-analysis, Binder et al. (2009) found that the most consistent semantic activation across 120 functional neuroimaging studies was located within the left angular gyrus (Binder et al., 2009). In addition, semantic processing can be perturbed after TMS stimulation or even impaired in patients with damage to the left IPL, suggesting a potential role for left IPL in controlled semantic processing (e.g., Jefferies and Lambon Ralph, 2006; Corbett et al., 2009; Whitney et al., 2011, 2012). We have also argued previously that the overlap between the semantic system and the DMN in the left angular gyrus was consistent with top-down “semantic constraints” during language comprehension (Price, 2010; Seghier et al., 2010).

In contrast, in anterior PCC and MPFC, deactivation was greater during semantic than perceptual matching. This particular pattern could not be solely attributed to task difficulty as deactivation in PCC and MPFC was not influenced by response times within tasks or by stronger demands on visual attention during perceptual decisions relative to saying “1,2,3” to the same stimuli (Table 1). In a previous meta-analysis, both regions, in particular the MPFC at the level of the ventral anterior cingulate cortex, have been related to action preparation, emotion, and interception (see Figure 2 in Laird et al., 2009). Recently, these two anterior subdivisions have also been associated with the valuation of highly salient (or personal) information, auto-biographical memory, and self-referential processing (for review, see Andrews-Hanna, 2012), and may be involved in both internal thoughts and external unfocused attention (Stawarczyk et al., 2011). Moreover, the two anterior regions in the PCC and MPFC were found to be the primary targets of incoming causal influences from other DMN regions (Jiao et al., 2011). In this context, we can speculate that when the brain is engaged in “external” processes that place strong demands on semantic processing, like our semantic matching and object naming tasks, the regions that are highly engaged in the generation of “internal” broad thoughts would be strongly deactivated/suppressed in order to focus the semantic system toward the external salient information. From a network perspective, using resting-state connectivity over more than 1000 subjects, Yeo et al. (2011) showed that both subdivisions in the MPFC and PCC were strongly connected to IPL (e.g., Figures 30 and 31 of Yeo et al., 2011), which may suggest that they also strongly interact during task-related processes. Therefore, the most likely interpretation of our findings is that, during semantic processing, activation is strongly suppressed in anterior PCC and anterior MPFC to focus attention on stimulus specific semantic processing in the left IPL.

### Impact of perceptual processing in the posterior PCC

Perceptual processing, relative to speech production in response to the same unfamiliar stimuli, was associated with greater deactivations in the posterior part of the PCC. This effect can be explained in terms of task difficulty because deactivation in posterior PCC was proportional to the number of errors made in each condition (Figures 1B and 4). An effect of task difficulty in posterior PCC corroborates a previous fMRI study that showed deactivation in PCC varied linearly with task difficulty, with greater suppression for the most error-prone conditions (Singh and Fawcett, 2008). Correlations between deactivation in PCC and task difficulty have also been reported in other studies (e.g., Mayer et al., 2010; Harrison et al., 2011; Leech et al., 2011), suggesting a potential role for this region in managing the allocation of attentional resources that increase with task demands. Our results are also in line with those of Lin et al. (2011) who showed that, using a calibrated fMRI method to quantify metabolic and hemodynamic changes within the DMN regions, greater effort was associated with greater deactivation in PCC but not MPFC (Lin et al., 2011). In contrast, other studies found that deactivation in both PCC and MPFC was sensitive to difficulty during auditory target decision (McKiernan et al., 2003) and visual motion discrimination (Singh and Fawcett, 2008). Therefore, the effect of difficulty is likely to depend on the type of task demands.

### Speech production in the posterior MPFC

Deactivations in the posterior-ventral MPFC showed a significant task effect, with greater deactivations during speech production tasks than matching tasks irrespective of stimuli. As far as we know, this is the first demonstration of task-induced deactivations in the MPFC that varied between language production tasks versus matching tasks when stimuli were held constant. More specifically, activation in the anterior cingulate cortex at similar coordinates to the area we report was observed for covert versus overt speech and paced versus unpaced speech production (see Figure 3 of Basho et al., 2007). This was interpreted in terms of sustained attention, motor planning, and response inhibition; which may also explain the response of this region in our paradigm because our subjects had to pace their production with the inter-stimulus interval.

Alternatively, other studies have suggested that MPFC might be related to working memory load rather than attentional demand (Mayer et al., 2010) and MPFC deactivation was also found to depend on the allocation of attentional resources when there were changes between task preparation and execution (Koshino et al., 2011). Accordingly, it is plausible that differences in terms of memory load, task preparation, and execution varied between our semantic matching and speech production tasks. Indeed, semantic matching involves both a search for semantic features that are shared across two stimuli, and short term memory to maintain these features while a decision is made, whereas naming and reading involve the retrieval of a unique conceptual representation that can be used to access the corresponding articulations of the associated words. Thus, these different processes between the two tasks may have significantly modulated the deactivation in the posterior-ventral MPFC.

### Picture processing in the right IPL

Greater deactivation during the processing of pictures of objects than their written names was only significant in an anterior part of the right IPL. In an early PET study (Bookheimer et al., 1995), greater deactivations during object naming than word reading were also observed in the IPL although the location of this effect in the anterior supramarginal gyrus (see Table 2 in Bookheimer et al., 1995) was slightly anterior to the region we report here (see Figure 4). Stronger right IPL deactivation for pictures could not be explained by task demands because it was observed during semantic matching when accuracy and RTs were comparable for pictures and words. Nor could it be explained by phonological processing (Petersen and Fiez, 1993) because deactivations were similar for reading aloud and perceptual matching on unfamiliar stimuli, even though these tasks vary in their demands on phonological processing. It is also unlikely that the right IPL is related to semantic processing because it was observed in an anterior region of IPL that was not sensitive to the semantic demands of the task (Figure 2A). Nevertheless it is possible that accessing the meaning of pictures relies on stronger links between visual inputs and semantics (Glaser and Glaser, 1989) which increases activation in left IPL (see review in Binder et al., 2009; Price, 2010; Seghier, 2012) while decreasing activation in right IPL. In this case, the deactivation in right IPL might be driven by homotopic inhibition from the left IPL. Future studies are required to test this hypothesis by assessing the coupling between left and right IPL during semantic processing on pictures versus words (for a similar rationale, see Carreiras et al., 2009; Seghier et al., 2011).

### Functional specialization within the DMN

Previous studies have segregated the DMN into different components (e.g., Laird et al., 2009; Margulies et al., 2009; Seghier and Price, 2009; Andrews-Hanna et al., 2010; Kim, 2010; Mayer et al., 2010; Leech et al., 2011; Stawarczyk et al., 2011; Andrews-Hanna, 2012). For instance, Leech et al. (2011) segregated the PCC during rest and an n-back working memory task into two subdivisions at (2 −58 28) and (2 −34 40) that are very close to our two PCC regions. In the same way, the two regions of the MPFC identified here are similar to subdivisions within the anterior cingulate cortex that were segregated according to their functional specialization and anatomical connectivity (see Beckmann et al., 2009). Other studies have segregated the same PCC and MPFC subdivisions using cytoarchitectonic techniques (e.g., Vogt et al., 1995, 2006; Ongür and Price, 2000; Ongür et al., 2003) or resting-state connectivity analyses (e.g., Uddin et al., 2009; Yeo et al., 2011). There is also growing evidence that each subregion in the DMN may sustain a specific functional role (e.g., Beckmann et al., 2009; Laird et al., 2009, 2011; Smith et al., 2009; Spreng et al., 2009; Kim, 2010) although the nature of each functional role varied across studies depending on the cognitive domain of interest (see review in Buckner et al., 2008; Kim, 2010; Andrews-Hanna, 2012). It may therefore be more relevant to define function at the level of co-ordinated networks of regions. In this context, our study highlights that, even in the language domain, (de)activation differs in different parts of the DMN depending on the exact demands of the language task. The exact function of each region, or network, remains an important endeavor for future studies.

## Conclusion

Our findings illustrate the strong functional heterogeneity across core DMN nodes during a range of language tasks. It is particularly relevant to language studies to note that the modulation of fMRI responses by semantic processing is not restricted to task-induced positive activations – semantic processing also enhances deactivations in discrete subdivisions of the DMN. The interplay between both positive and negative task-related responses is crucial for the success of a given process/task. In this context, it is not surprising that the failure to (de)activate the DMN has been shown to be a reliable marker of abnormal cognition across a wider spectrum of mental diseases (e.g., Kennedy et al., 2006; Pomarol-Clotet et al., 2008). Future studies are needed to assess how these subdivisions are interconnected and how they interact with other task-activated regions of the language system during rest.

## Conflict of Interest Statement

The authors declare that the research was conducted in the absence of any commercial or financial relationships that could be construed as a potential conflict of interest.
